# Flow and wake characteristics associated with large wood to inform river restoration

**DOI:** 10.1038/s41598-021-87892-7

**Published:** 2021-04-21

**Authors:** Isabella Schalko, Ellen Wohl, Heidi M. Nepf

**Affiliations:** 1 Department of Civil and Environmental Engineering, Massachusetts Institute of Technology, Cambridge, MA USA; 2 Laboratory of Hydraulics, Hydrology and Glaciology, ETH Zurich, Zurich, Switzerland; 3 Department of Geosciences, Colorado State University, Fort Collins, CO USA

**Keywords:** Fluid dynamics, Geomorphology

## Abstract

Wood is an integral part of a river ecosystem and the number of restoration projects using log placements is increasing. Physical model tests were used to explore how the wood position and submergence level (discharge) affect wake structure, and hence the resulting habitat. We observed a von-Kármán vortex street (VS) for emergent logs placed at the channel center, while no VS formed for submerged logs, because the flow entering the wake from above the log (sweeping flow) inhibited VS formation. As a result, emergent logs placed at the channel center resulted in ten times higher turbulent kinetic energy compared to submerged logs. In addition, both spatial variation in time-mean velocity and turbulence level increased with increasing log length and decreasing submergence level. Submerged logs and logs placed at the channel side created a greater velocity deficit and a longer recirculation zone, both of which can increase the residence time in the wake and deposition of organic matter and nutrients. The results demonstrate that variation in log size and degree of submergence can be used as a tool to vary habitat suitability for different fish preferences. To maximize habitat diversity in rivers, we suggest a diverse large wood placement.

## Introduction

**Figure 1:**
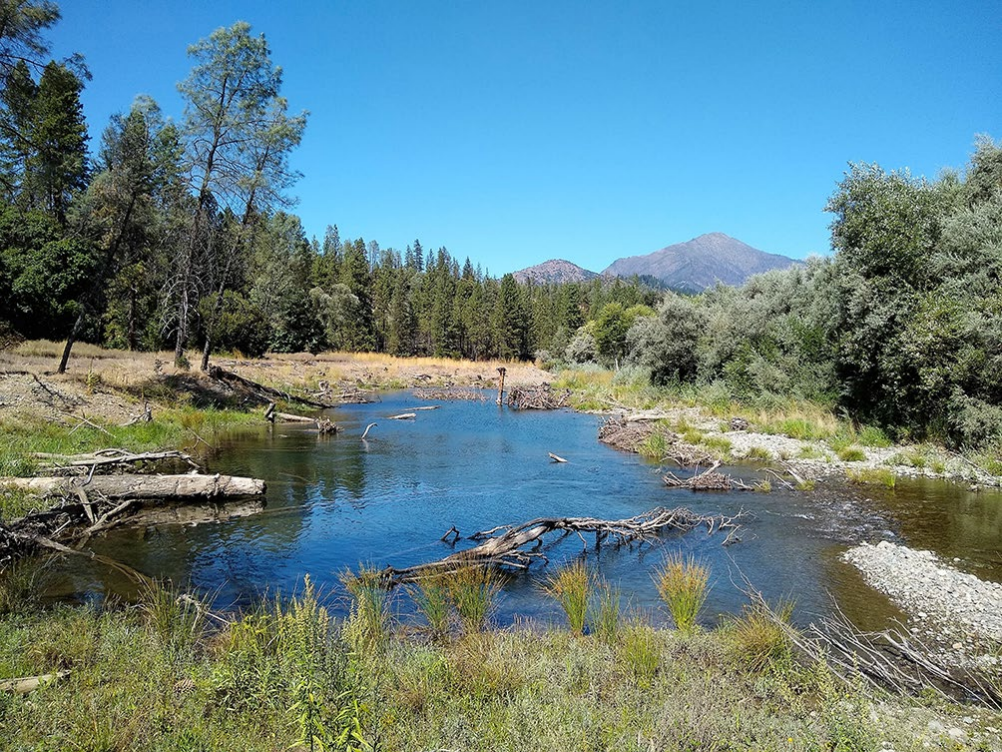
Side channel with wood placements at the Trinity River, California, USA (photo: I. Schalko).

Over the past centuries, human interventions have significantly impacted the characteristics of rivers, such as flow regime or channel geometry^1^. Due to these actions, many rivers worldwide exhibit sediment deficit as well as hydraulic and morphological degradation^2,3^. The imbalance of flow, sediment, and wood discharge has severe effects on the river ecosystem such as loss of fish spawning habitat or channel incision^1,4^. The restoration of river habitat has become a critical problem throughout the world, with prominent activities in Europe, Australia, and the USA, representing projects worth billions of US dollars^5,6^. The majority of river projects focus on restoring the flow and sediment regimes^4^. However, consideration of the natural wood regime is also crucial, because wood provides habitat and contributes to nutrient cycling by trapping fine material^4^. The number of river restoration projects including wood has increased within the past decades^7,8^, as the perception of wood in rivers is shifting from a hazard towards a beneficial and valuable part of a river ecosystem^7,9,10^. Wood plays an essential role for a river ecosystem, as it can create heterogeneous flow conditions and morphological structures^11,12^. Individual pieces (Fig. 1) through large accumulations of multiple pieces can create different, distinctive, local- to reach-scale hydraulic and geomorphic features^13^. Similar to a vegetation patch or boulder^14^, ecologically beneficial dead-water zones, a region characterized by low flow velocity and reduced mixing, may form up- or downstream of logs, in which nutrients, organic matter, or sediments can be deposited^15–19^. In addition, wood increases the vertical connectivity and residence time of hyporheic exchange flow^20,21^ and provides habitat for fish^7,22,23^.

A common restoration method using wood is an engineered log jam (ELJ), defined as a groyne-like structure installed to alter the flow, protect the river banks^24^, or stabilize an incised channel^25^. Design guidelines on ELJs have been summarized in several publications (Brooks^24^, Abbe and Brooks^26^, USBR & ERDC^8^), with special focus on Australia and the US Pacific Northwest. The majority of previous studies focused on emergent ELJs with different designs^27,28^, discharge conditions^29^, and porosity^30^. For ELJs with increasing porosity, the flow passing through the ELJ into the downstream wake (bleed flow) reduced the downstream turbulence levels and scour^29,30^. Emergent ELJs located at the channel wall created reduced velocity just downstream of the structure, a lateral mixing layer extending about one-half of the structure’s width into the flow, and enhanced velocity at the center of the channel, extending to the opposite bank^27^. Individual logs that span the channel or logjams of multiple logs are also used in restoration and may induce an increase in upstream water depth (backwater rise) due to the blocked flow cross-section. As the backwater effect is proportional to the obstruction ratio, defined as the ratio of log dimensions with respect to the channel width and flow depth^31,32^, an emergent logjam will lead to larger backwater rise and pool formation. Most logjams have some flow through or below them, although some jams are effectively impermeable and have flow only over the top^33^.

To plan and evaluate river restoration projects that aim to create habitat for fish, it is crucial to anticipate the changes in flow velocity and turbulence characteristics. Smith et al.^34^ hypothesized that fish select their habitat based on turbulence attraction and avoidance. Fish sense increased turbulence due to flow separation and use this to locate roughness elements such as wood for cover (i.e., attraction)^34^. Within the wake of wood, fish then select a region with lower turbulence level (i.e., avoidance)^34^, which can provide velocity shelters^34,35^. Other studies on fish locomotion demonstrated that fish seek areas of increased turbulence to reduce locomotory costs^35–37^ and alter their body kinematics to match the vortex shedding frequency of a structure^37,38^. According to Tullos and Walter^39^, wood can establish both regions of reduced velocity (wake), which provide shelter for resting, and also regions of increased velocity (next to wood placement), which provide higher drift densities for more efficient feeding and higher rates of energy gain. However, flow characteristics such as vorticity and eddy length can also affect the swimming capabilities of fish. Fish tend to lose their postural control when the length-scales of shear and turbulent eddies are comparable to the body dimensions of the fish^40,41^. The flow around a single log is similar to flow around a bluff body, producing a wake with reduced velocity and transverse shear that may enhance turbulence production at the scale of the body.

Previous studies on engineered wood for restoration have mainly focused on emergent ELJs. In prototype, a wood placement may be submerged during higher discharges, while being emergent during lower discharges. When a log is submerged, flow passing over the log and into the wake (sweeping flow) may suppress the formation of the von-Kármán vortex street (VS), as observed for submerged vertical cylinders^42^, groynes^42^, porous vegetation patches^43^, and porous ELJ^30^. The magnitude of sweeping flow will depend on both the degree of submergence and the length of the log (cross-channel length-scale), but these dependencies have not yet been defined. The suppression of the VS will have implications for the extent of the recirculation zone, magnitude of turbulent kinetic energy, and spatial footprint of reduced velocity in the channel. The flow structures governing fish behavior are known, but knowledge is missing on the flow and wake structures of different wood placements. Therefore, we conducted physical model tests to study the flow and wake structures due to different wood placements on a solid bed. The time-mean velocity, turbulent kinetic energy, and turbulence integral length-scale were analyzed for various wood placements and relative submergence levels and compared to the approach flow condition. The different wood placements were characterized by different log lengths, log diameters, log orientation relative to the flow, and location of wood placement at the channel center versus at the channel sidewall. The experiments presented here considered individual logs that partially spanned the channel, thereby representing a sub-set of natural and engineered conditions. The results from physical model tests were compared to field observations. Finally, the implications for fine particles deposition and fish habitat creation were considered.

## Method

**Figure 2:**
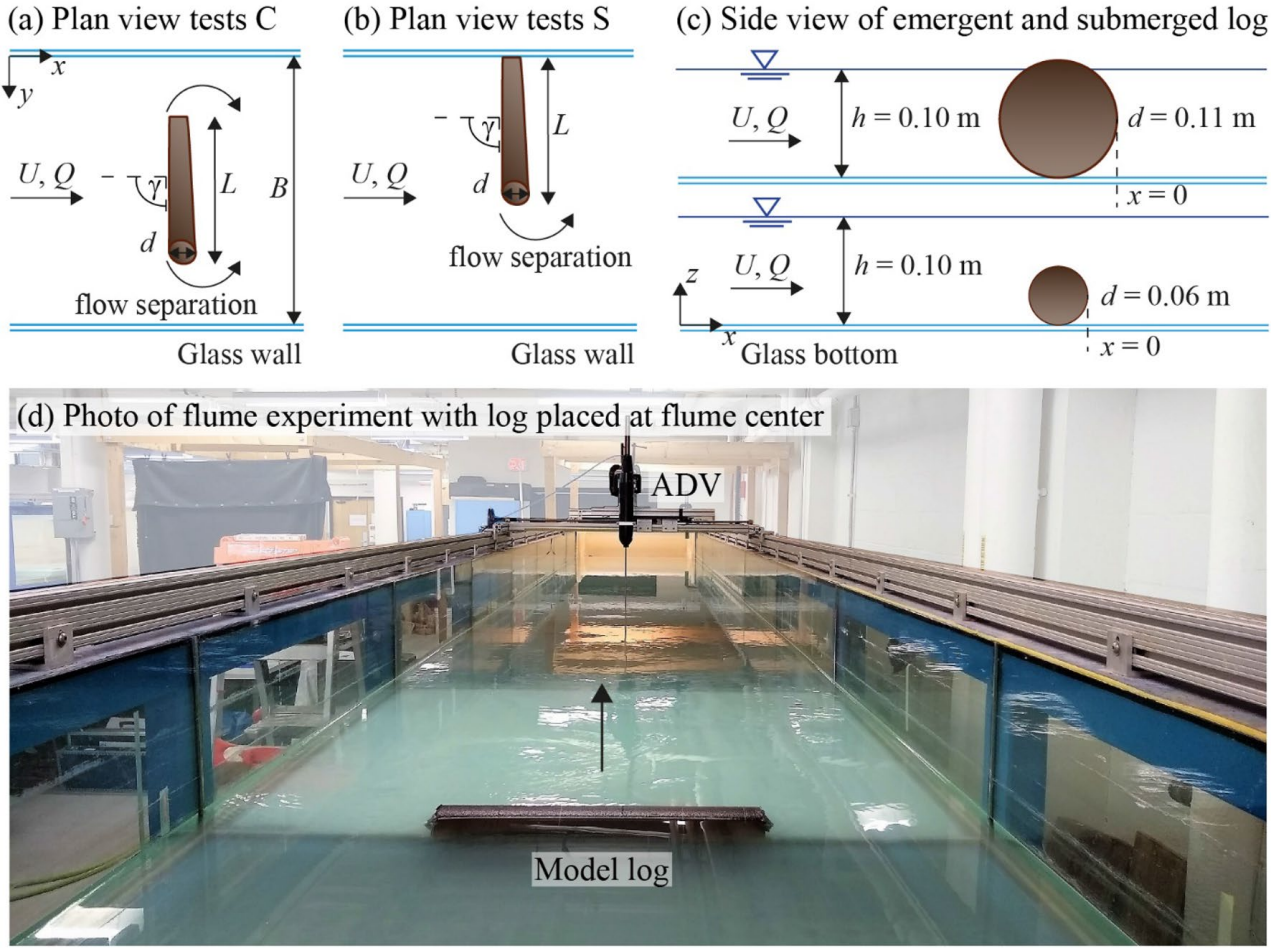
(**a**) Plan view of test setup with log positioned at channel center, denoted by “C” and (**b**) side, denoted by “S”, with *U* = cross-sectional averaged flow velocity, *Q* = discharge, *d* = log diameter, *L* = log length, *γ* = log orientation angle, *B* = channel width, and *h* = flow depth; (**c**) side view of emergent and submerged log; (**d**) photo of flume experiment looking downstream. Note that *x* = 0 was defined at the downstream trailing edge of the log.

The flume experiments were conducted at the Massachusetts Institute of Technology in a 12.2 m long, 1.2 m wide, and 0.70 m deep glass-walled channel with a fixed bed. The test setup and notation are illustrated in Fig. 2. The flow discharge *Q* was controlled by a variable-speed pump and measured with a flow meter. The cross-sectional averaged velocity was defined as *U* = *Q*/(*B h*) with *B* = channel width, and *h* = flow depth. The approach flow (AF) conditions were measured 1 m upstream of the log placement. A single log was modeled using a PVC pipe with a roughness coat (equivalent sand roughness in prototype of *k*_*s*_ = 5 mm) to imitate bark. The log diameters were *d* = 0.06 m, 0.09 m, and 0.11 m, and the log lengths were *L* = 0.13 m, 0.25 m, 0.50 m, and 0.75 m. The log was placed at the channel center (Fig. 2a) and at the side (Fig. 2b). The log orientation angles to the flow in the horizontal plane were *γ* = 90° (perpendicular to the flow), 45°, and 30°. The experiments were designed based on both Reynolds number (R = *U h/v*, R_*L*_ = *U L/v*) and Froude number (F = *U*/(*g h*)^0.5^) similitude, with *g* = gravitational acceleration. Specifically, R = 10^4^ and R_*L*_ > 10^4^, ensuring fully turbulent flow and turbulent wake structures.

The flow velocity (streamwise *u*, lateral *v*, and vertical *w*) was measured along vertical, lateral, and longitudinal profiles up- and downstream of the log using Acoustic Doppler Velocimetry (Nortek Vectrino). A downward looking probe was used with 200 Hz sampling rate measured for a duration of 240 s to reach convergence. The velocity records were despiked and filtered according to Goring and Nikora^44^. Each velocity record was decomposed into the time-mean $\left( {{\overline{u}},{\overline{v}},{\overline{w}}} \right)$ and fluctuating (*u*′, *v*′, *w*′) components using a MATLAB script. The turbulent kinetic energy was

1 $${k_t} = (\overline{{u}^{{\prime}{2}}} + \overline{{v}^{{\prime}{2}}} + \overline {{w}^{\prime}{2}} {)/2}{\text{.}}$$

The longitudinal profile at log center was used to evaluate the location of the minimum and maximum value of ${\overline{u}}$ and *k*_*t*_ and to characterize the wake and recirculation zone dimensions.

The wake length *L*_*w*_ was defined as the distance from the trailing edge of the log to the position at which ${\overline{u}}$ = *U* or *d* ${\overline{u}}$/*dx* = 0 (± 10%). The length of the reattachment zone *L*_*r*_ was set as the distance from the log trailing edge to the position at which the recirculating flow recovered and ${\overline{u}}$ ≥ 0. For selected tests, dye was added to the flow to visualize the vortex structures. The integral length scale *L*_*x*_ was determined from the autocorrelation function of the local streamwise velocity at multiple positions downstream of the log. For this method, only positions for which ${\overline{u}}$ > 0 (i.e., not in a recirculation zone), and turbulence strength *u*_*rms*_ small compared to ${\overline{u}}$ could be considered (see details in Supplementary Information). For tests with an observed VS, the integral length scale Λ_*peak*_ associated with the peak frequency *f*_*peak, v*_ of the power spectra of the lateral velocity component *S*_*vv*_ was also estimated, as this reflects the size of the coherent VS structures. The procedures to determine the integral length scales are described in detail in the Supplementary Information. The test program is summarized in Table 1. Experiments with the log at the channel center (C1–C7) were conducted for emergent logs with various lengths (C1e–C3e, *L*), and for submerged logs with two diameters (C4s–C5s, *d*) and different log orientations to the flow (C6e–C7e, *γ*). The experiments with the log placed at the channel side (S1–S7) were performed for emergent logs (S1e–S3e) and submerged logs (S4s–S7s) with various lengths.

**Table 1:** Test program with flow depth *h* = 0.10 m and cross-sectional average velocity *U* = 0.10 m/s (C = log at center, S = log at side, e = emergent, s = submerged).

Test	Discharge	Log length	Log diameter	Relative submergence	Log angle	Wake length	Reattach-ment length	TKE length	Integral length scale	
#	*Q* (l/s)	*L* (m)	*d* (m)	*h/d* (–)	*γ*_*L*_ (°)	*L*_*w*_ (m)	*L*_*r*_ (m)	*L*_*TKE*_ (m)	$\overline{{\Lambda_x}}$(m)	$\Lambda_{peak}$(m)
C1e	12.72	0.75	0.11	0.91	90	3.50	–	2.00	1.3 ± 0.7	0.77
C2e	12.72	0.50	0.11	0.91	90	2.00		1.50	0.16 ± 0.08	0.46
C3e	12.72	0.25	0.11	0.91	90	2.50		0.23	0.04 ± 0.01	0.20
C4s	12.72	0.50	0.09	1.63	90	4.50		0.27	0.08 ± 0.05	–
C5s	12.72	0.50	0.06	1.17	90	6.00		0.19	0.07 ± 0.07	
C6s	12.72	0.50	0.09	1.17	45	5.50		0.18	0.06 ± 0.03	
C7s	12.72	0.50	0.09	1.17	30	3.50		0.27	0.04 ± 0.04	
S1e	12.72	0.50	0.11	0.91	90	–	6.50	5.00	0.23	–
S2e	12.72	0.25	0.11	0.91	90		3.50	3.50	0.23 ± 0.10	
S3e	12.72	0.13	0.11	0.91	90		1.50	1.50	0.09 ± 0.07	
S4s	12.72	0.50	0.09	1.17	90		3.00	0.27	0.05 ± 0.04	
S5s	12.72	0.25	0.09	1.17	90		2.50	0.18	0.11 ± 0.08	

The measured wake length *L*_*w*_ and reattachment length *L*_*r*_ exhibit a consistent uncertainty of *Ο*(*L*) due to measurement spacing. Longitudinally averaged $\Lambda_x$ with standard deviation (except for S1e, which is a single measurement point). For cases with a VS (C1e–C3e), the peak integral length scale $\Lambda_{peak}$ is also listed.

### Approach flow characteristics

**Figure 3:**
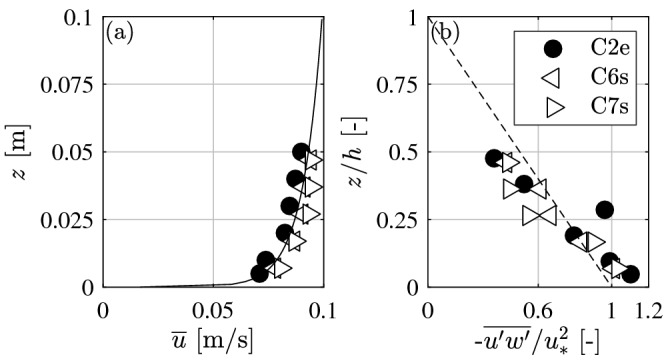
Vertical profiles of (**a**) approach flow velocity (solid line represents logarithmic law, Eq. 2) and (**b**) relative turbulent shear stress (dashed line represents linear profile, Eq. 3).

The approach flow was defined 1 m upstream of the log. The vertical profiles of time-averaged velocity ${\overline{u}}$ followed the logarithmic distribution characteristic for quasi-uniform open-channel flows (Fig. 3a)

2 $$\bar{u}\left( z \right) = \frac{{2.3u_{*} }}{\kappa }\log _{{10}} \left( {\frac{z}{{z_{0} }}} \right) ,$$

with u_*_ = shear velocity, *z*_0_ = characteristic roughness, and κ = 0.40 is the von-Kármán’s constant. Both u_*_ and *z*_0_ were estimated by fitting the velocity profiles using Eq. (2). In addition, the measured vertical profiles of the turbulent shear stress $- \overline{{u^{{\prime }} w^{{\prime }} }}$ (Fig. 3b) follow the expected linear trend

3 $$- \overline{{u^{{\prime }} w^{{\prime }} }} = u_{*}^{2} \left( {1 - \frac{z}{h}} \right).$$

The value for u_*_ was estimated by fitting the turbulent shear stress profiles using Eq. (3). For the subsequent analysis, u_*_ was determined by averaging the results of Eqs. (2) and  (3) with u_*_ = 0.0039 m/s. Based on u_*_ and *U* we can define the quadratic-law bed friction parameter *C*_*f*_ = 2 (u_*_/*U*)^2^ = 0.0035.

## Results and discussion

### Wake characteristics and spectral analysis

**Figure 4:**
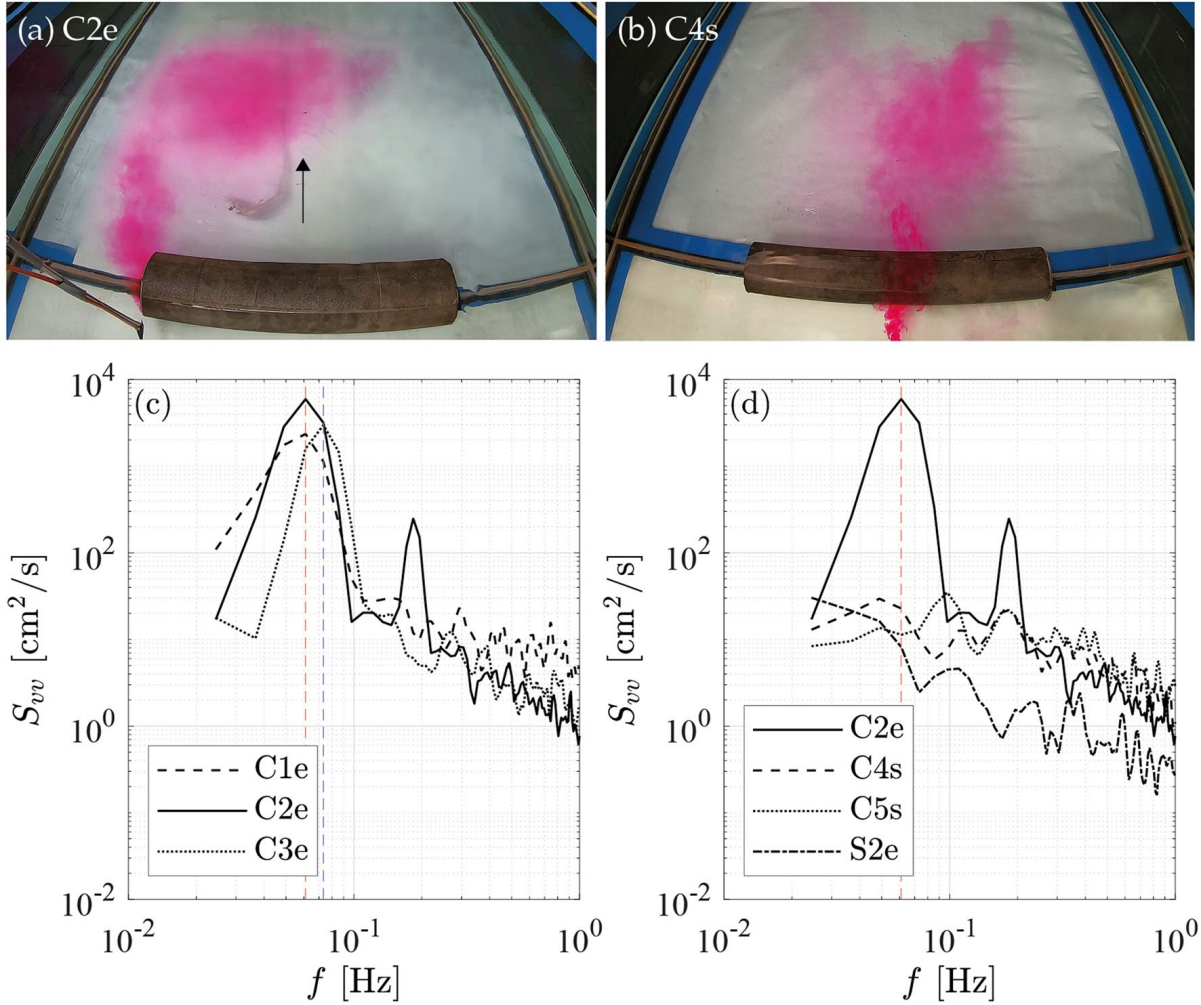
(**a**) and (**b**) Photos of C2e with 50-cm long emergent log and C4s with 50-cm long submerged log with tracer injected upstream of log to illustrate the presence (**a**) and absence (**b**) of a von-Kármán vortex street. When present, the vortex street was unsteady, with vortices forming and shedding from either end of the log in a regular alternating pattern. (**a**) illustrates the formation of a single vortex on the left-side of the log, and subsequently a vortex forms on the right-hand side; (**c**) and (**d**) Power spectra of lateral flow velocity component measured at log center at maximum *k*_*t*_*.*

For an emergent log, the approach flow was deflected laterally around the log, creating a lateral gradient in velocity, i.e., a shear layer, at the ends of the log. Downstream of the log, a turbulent wake was generated, including a recirculation zone. For emergent logs placed at the channel center, a von-Kármán vortex street (VS) was formed, characterized by the periodic detachment of vortices from alternating ends of the log (Fig. 4a; note that only one vortex is illustrated as the dye was injected on one side). The vortex street results from an unstable interaction of the shear layers generated at either end of the log. In contrast, no VS was formed by a log placed at the channel side, because it generated a single shear layer, for which the shear-layer instability leading to a VS does not occur. In addition, no VS was formed for submerged logs, due to the suppression of vortex shedding by the flow passing above the log and into the wake (sweeping flow, Fig. 4b). This has also been observed for submerged vertical cylinders (with the relative submergence defined as flow depth *h* to cylinder height *h*_*c*_) with 1.8 < *h*/*h*_*c*_ ≤ 4, while a VS was formed for slightly submerged and emergent cylinders with 0.7 < *h*/*h*_*c*_ ≤ 1.1^45,46^. For emergent obstacles (*h*/*h*_*c*_ = 1) with low aspect ratio, i.e., cylinder height to width near one, the shape does not affect the wake structure, whereas shape is important to submerged obstacles (1 < *h*/*h*_*c*_ < 5.5)^47^. Considering a log, the aspect ratio is defined as the log diameter to log length, resulting in very low aspect ratios (*d*/*L* = 0.09, 0.12, and 0.15) that significantly differ from classic cylinder studies.

The presence/absence of the VS was also confirmed with the power spectra of the lateral velocity component *S*_*vv*_ (Fig. 4c,d). Spectra were evaluated at the location of maximum *k*_*t*_ at the log center. For all emergent logs placed at the channel center there was a peak in *S*_*vv*_ at frequency *f* = 0.06 to 0.07 Hz (Fig. 4c; C1e–C3e), confirming the presence of VS. The log Reynolds number was R_*L*_ = *U* *L*/ν = 1.2 to 7.0 × 10^4^ for all tests. For the shortest emergent log, C3e, with *L* = 0.25 m, *f* = 0.07 Hz, resulting in Strouhal number *St*_*L*_ = (*f L*)/*U* = 0.18. This was similar to the vortex shedding of a vegetation patch^43^ or an unconfined solid cylinder^48^. For the two longer logs (0.5 and 0.75 m), the observed Strouhal numbers (0.31 and 0.46, respectively) were higher than the unconfined value (0.18). Based on previous studies, this was attributed to the high flow blockage in these cases, (*L*/*B* = 0.42 and 0.63). Previous studies have shown that when a shedding structure is confined by walls, velocity enhancement adjacent to the structure increases in shedding frequency, i.e., increases the Strouhal number^49,50^.

In contrast to the emergent, centered logs, there was no peak in *S*_*vv*_ for either the submerged logs at the channel center or the emergent logs at the channel side (Fig. 4d; C4s, C5s, S2e). The suppression of the VS was not associated with bed friction. In shallow flow, a wake can be stabilized by bed friction, *C*_*f*_, if the wake stability parameter *S* = *C*_*f*_* L/h* > 0.2^50^. In our experiments, *S* = 0.0043 to 0.026, which was significantly smaller than the critical values defined by Chen and Jirka^51^. Therefore, the bed friction was not large enough to suppress the vortex shedding process. Instead, the fact that a VS was not observed behind the submerged logs was attributed to the presence of flow over the log that entered the wake (sweeping flow) and suppressed the interaction of the two shear-layers, thus suppressing the formation VS (see discussion in Zong and Nepf^43^). The log placed at the side generated only one shear layer, which does not produce a VS.

It is useful to consider whether bed friction could influence wake structure in the field. Using a scale factor of λ = 5, the flow depth *h* and log lengths *L* of the flume experiments were upscaled to field conditions (subscript *f*) to *h*_*f*_ = 0.50 m and *L*_*f*_ = 1.25, 2.5, and 3.75 m. Using the critical stability parameter *S*_*c*_ = 0.2, the critical bed friction required to suppress VS was determined to be *C*_*f*_ = 0.08, 0.04, 0.03 for *L*_*f*_ = 1.25, 2.5, 3.75 m, respectively. The sediment size *d*_*s*_ producing these bed friction coefficients was derived by applying the semi-empirical equation in Julien^52^:

4 $$C_f{ = }\frac{{1}}{{\left[ {{5}{\text{.75log}}\left( {\frac{{{\text{2}h}}}{{{{d_s}} }}} \right)} \right]^{{2}} }},$$

which resulted in *d*_*s*_ = 0.25, 0.14, 0.09 m for *L*_*f*_ = 1.25, 2.5, 3.75 m, respectively (*S*_*c*_ = 0.2), i.e., to suppress the VS the grain size must be of the scale of large gravel or greater.

Turbulent structures within the wake may impede the swimming capabilities of fish, if the size of the eddy is in the range of the fish length^40,41,53^. For emergent logs positioned at the centerline, which generated a VS, the most meaningful turbulence length-scale is that of the VS coherent structures, described by $\Lambda_{peak}$. As expected for a VS, $\Lambda_{peak}$ scaled with log length, and specifically, $\overline{{\Lambda_{peak}}} /{L}$ = 0.92 ± 0.09 (SD, see Table 1). The scale $\Lambda_{peak}$ ≈ $L$ was also illustrated with tracer, which visualized the VS eddy (Fig. 4a). For submerged logs at the channel centerline, which did not form a VS, the eddy size was significantly reduced compared to the emergent logs and scaled with the water depth. Averaging across all measurements in submerged-center-log placements (C4, C5, C6, C7), ${ }\overline{{\Lambda_x}}$ = 0.06 ± 0.04 m (SD across all measurements). For the side placements, the eddy-scale behind an emergent log, $\overline{{\Lambda_x}}$ = 0.18 ± 0.09 m was again larger than those behind a submerged log, $\overline{{\Lambda_x}}$ = 0.08 ± 0.06 m. The key points were that for emergent logs, eddy size can depend on log length, and that the eddy size decreased significantly when logs transitioned from emergent to submerged.

**Figure 5:**
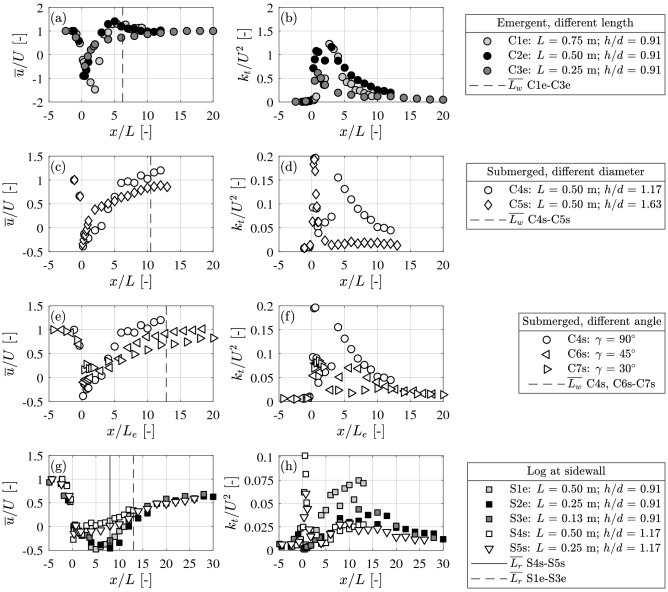
Longitudinal profiles of normalized time-mean velocity and turbulent kinetic energy along log centerline. (**a**), (**b**), and (**c**) logs positioned at the channel center and (**a**) emergent logs of different length, (**b**) submerged logs of different diameter and relative submergence, *h*/*d*, (**c**) submerged logs with different log angle. *L*_*e*_ is the projected log length. (**d**) Emergent and submerged logs positioned at the sidewall. Vertical lines mark the average wake length, ${{L_w}}$, for tests C1-7 and average length of reattachment zone, ${{L_r}}$, for tests S1-5.

Longitudinal profiles of ${\overline{{u}}}$ and *k*_*t*_ were measured at the midpoint of log length and at *z* = *d*/2 (Fig. 5). The downstream trailing edge of the log was at *x*/*L* = 0. Compared to the approach flow (measured 1 m upstream of the log), normalized velocity, ${\overline{{u}}}$/*U,* decreased, whereas normalized *k*_*t*_/*U*^2^ increased in the log wake (Fig. 5). Similar to previous studies, log placement increased the spatial variability in the flow^27,28^, but our results also demonstrated that variation in log size and positioning can significantly alter the velocity and turbulence in the wake, in some cases by an order of magnitude. As log length (*L*) increased, the minimum velocity in the wake *u*_min_ decreased, but *k*_*t*_ increased (Fig. 5a,b). For *L* = 0.75 m, *u*_min_ was located *x*/*L* = 2 downstream of the log and for *L* = 0.25 m and 0.50 m (C3e and C2e) at *x*/*L* = 0.5. For submerged logs, *u*_min_ was two-times larger than that observed for emergent logs with the same *L* (C2e compared to C4s and C5s; Fig. 5c). For logs at the channel center, the wake length *L*_*w*_ scaled with the log length and was longer for submerged logs with *L*_*w,s*_ = (9 to 15) *L* compared to *L*_*w,e*_ = (4 to 10) *L* for emergent logs (Fig. 5a,c,e; Table 1). The slower recovery of velocity (longer *L*_*w*_) for submerged logs can be attributed to the weaker turbulence in the wake of a submerged versus an emergent log (compare Fig. 5b,d). Turbulence was weaker in the wake of the submerged logs because the flow passing over the log and into the wake suppressed the production of VS (Fig. 4d). Specifically, for log length *L* = 0.50 m, *L*_*w*_ was 1.6 (± 0.5) times longer for submerged compared to emergent logs. The longer wake for submerged logs (Fig. 5c) can provide a longer downstream region that promotes the deposition of organic matter and nutrients^15,19^ in contrast to the shorter, more energetic wake for emergent logs.

The degree of log submergence impacted both the position and magnitude of peak turbulence. For the emergent cases (*h*/*d* < 1), the distance from the log to the point of maximum *k*_*t*_ scaled with *L*, which was associated with the observed formation of the VS. Specifically, peak *k*_*t*_ occurred at (2.2 ± 1.1) *L* (Table 1). Because of the coherent VS, the turbulence in an emergent log wake was 10 × higher than that in a submerged log wake (compare 5b and 5d). For submerged cases, the distance to the maximum *k*_*t*_ scaled with *d*, associated with the vertical recirculating eddy formed directly downstream form the log. Specifically, peak *k*_*t*_ occurred at ≈ 3 *d* for all submerged log lengths (Table 1). For the deeper submergence, *h*/*d* = 1.63, the flow over the log (sweeping flow) entered the wake, disrupting the interaction between the two shear layers, thereby suppressing the VS, which resulted in low turbulence downstream of the first peak (Fig. 5d). In contrast, for mild submergence, *h*/*d* = 1.17, a hybrid case with two peaks in turbulence was observed. The first peak was directly behind the log and at the same point as that observed for the higher submergence (≈ 3 *d*). The second, smaller peak was at 4 *L*, which was consistent with the emergent cases, suggesting this peak was associated with a weak VS.

The effect of the log angle γ on wake structure can be represented by the projected log length, *L*_*e*_ = sin(γ) *L*. Specifically, the wake-length scaled with *L*_*e*_, i.e., *L*_*w*_ = (12.8 ± 3.4) *L*_*e*_ (Table 1 and Fig. 5e,f). The angled logs were all submerged with *h*/*d* = 1.17 (C6s and C7s), for which two turbulence peaks were observed, consistent with case C4s (Fig. 5f). The magnitude of maximum *k*_*t*_ decreased with decreasing angle (γ) and *L*_*e*_ (C4s with *L* = *L*_*e*_ = 0.50 m versus C6s with *L*_*e*_ = 0.35 m).

Additional experiments were performed with the log at the channel sidewall, including both emergent (S1e–S3e) and submerged cases (S4s–S5s). The reattachment length *L*_*r*_ was defined as the distance at which the sidewall boundary layer reattached to the channel wall. For the side log placement, mixing between the outer flow and the wake was restricted to one side, and no VS was generated (Fig. 4d). As a result, logs placed at the channel side produced a larger and more pronounced recirculation zone and total wake length, compared to the center log placement. The greater length of recirculation can increase the residence time of organic matter and nutrients in the wake, enhancing deposition processes. For emergent cases, *L*_*r*_ was (13 ± 1) *L* (Fig. 5g; Table 1), which was similar to the range of reattachment lengths observed for emergent groynes, (11 to 17) *L*^54^. For the submerged cases, *L*_*r*_ was smaller than the emergent cases, with *L*_*r*_ = (8 ± 3) *L* (Fig. 5g; Table 1).

For logs placed at the side, the position of *k*_*t*_ was similar to that observed for logs placed at the channel center. Specifically, for submerged cases *L*_*TKE*_ scaled with the log diameter, *L*_*TKE*_ = (2.5 ± 0.7) *d*, and for emergent cases with the log length, *L*_*TKE*_ = (12 ± 2) *L* (Table 1). However, side log placement produced significantly weaker turbulence. Specifically, the peak *k*_*t*_ for sidewall placement was 10% and 50% of that observed for center-channel placement, for emergent and submerged conditions, respectively. Further, for the hybrid condition with two *k*_*t*_ peaks, the second peak was less pronounced for side log placement (S4s and S5s; Fig. 5h) compared to the center log placement (C4s; Fig. 5d).

The flume experiments were conducted with a fixed bed, comparable to an incised bedrock channel, thereby neglecting local suspended sediment and bedload transport processes associated with log placement in real rivers. Given a mobile bed, these processes are governed by the ratio of the shear velocity u_*_ to the sediment settling velocity *w*_*s*_ (suspended sediment) and the critical shear velocity u_*c_ (bedload). The observations described above for a fixed bed can be used to infer changes in log wakes when a mobile bed causes a log to become buried. For example, erosion around the log could decrease the log frontal area exposed to the flow, which in turn would affect the wake characteristics. For a submerged log, a decrease in exposed log diameter would increase the flow over the log (sweeping flow), suppressing the generation of the VS, resulting in weaker turbulent mixing, and an increase in wake length. Depending on the degree of erosion, an emergent log may become submerged, which would result in an increased wake length due to the sweeping flow. If the log remains emergent, the wake will not change, because the wakes of emergent logs are governed by the log length or projected length.

Changes in bed morphology can be anticipated based on the wake dynamics. For example, the increased shear at the log ends and the enhanced *k*_*t*_ downstream of an emergent center log (Fig. 5a,b) may produce scour to the sides and downstream of the log. In comparison, submerged logs would only create scour directly behind the log, associated with the plunging overflow, similar to step-pool channels^31,55,56^. In addition, the recirculation and velocity deficit zones associated with the submerged and side log placement would be regions of reduced u_*_, which would enable sediment deposition. According to field observations, backwater and scour effects tend to decrease with increasing submergence level and increase with increasing discharge^56–58^. The deposition of fine sediment in the backwater as well as the scour of sediment from the downstream plunge pool also decrease with submergence level and increase with discharge^57^. Recent flume and field studies on emergent side logs showed that while single logs increased erosion rates, logs placed in series did not increase erosion rates, as the wake interference between the logs reduced the near-bank velocity^59–61^.

**Figure 6:**
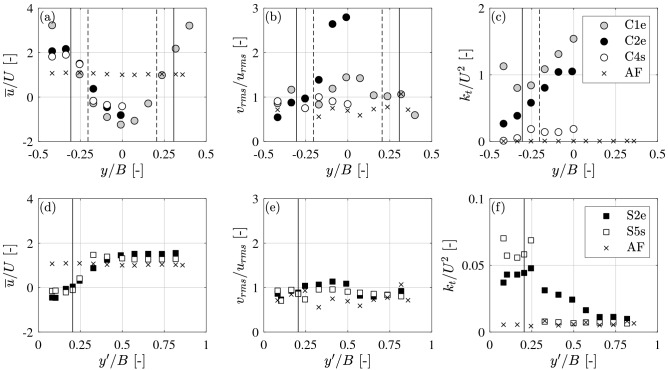
Lateral profiles of time-mean velocity normalized by the channel average velocity (**a**,**d**), ratio of *v*_rms_ to *u*_rms_ (**b**,**e**) and turbulent kinetic energy normalized by the square of channel average velocity (**c**, **f**) for logs placed at center (**a**–**c**) and at the side (**d–f**). Approach flow conditions (AF) included for reference; log center at *y/B* = 0 (placed at channel center) and channel wall at *y’/B* = 0 (placed at channel side). *B* is the channel width. Note that the range on the vertical axis in (**c**) is 20 times greater than in (**f**). The vertical lines indicate the log ends. For (**a**,**b**) and (**d**–**e**) (*u* and *v*_rms_/*u*_rms_), the lateral profile was taken at the *x*-location at which flow velocity was minimum. For (**c**) and (**f**) (*k*_*t*_), the *x*-location corresponded to the peak *k*_*t*_.

The spatial footprint of the wake and the influence of a VS were further illustrated by the lateral profiles of ${\overline{{u}}}$, relative turbulence strength *v*_rms_/*u*_rms_, and *k*_*t*_ (Fig. 6). All measurements were made at *z* = *d*/2. For logs at the channel center, the lateral coordinate was zero at the channel and log centerline (*y/B* = 0). But for the logs placed at the channel wall, the lateral coordinate was zero at the wall (*y’/B* = 0). First, consider the velocity variation around the log. As the fraction of channel cross-section blocked by the log increased, the velocity enhancement around the side of the log also increased. For case C1e (emergent, center log, *L* = 0.75 m, grey dots) a full lateral profile was collected, which confirmed that the log center acted as a line of symmetry (Fig. 6a). To decrease the measurement effort, the subsequent measurements were made for just one side of the log. The increased velocity at the side of the log was greatest for the widest log, test C1e (*L* = 0.75 m), which reached ${\overline{{u}}}$/*U* = 3.2 compared to ${\overline{{u}}}$/*U* = 2.1 for the shorter log C2e (*L* = 0.5 m) and ${\overline{{u}}}$/*U* = 1.8 for the submerged log C4s (*L* = 0.5 m). In comparison, the increase in velocity around a log of the same length but placed at the channel side was smaller, reaching only ${\overline{{u}}}$/*U* = 1.6 for S2e (*L* = 0.25 m) and ${\overline{{u}}}$/*U* = 1.3 for S5s (*L* = 0.25 m, Fig. 6d).

Because the VS preferentially enhanced the lateral component of fluctuating velocity, the presence of the VS was correlated with an increase in lateral turbulence intensity, reflected in the ratio *v*_rms_/*u*_rms_ (Fig. 6b,e), and this ratio further illustrates the differences between the emergent center logs (with VS), and submerged and sidewall logs (without VS). For logs positioned at the channel center, the lateral profiles of normalized *k*_*t*_ (Fig. 6c) show that the peak turbulence level occurred at the centerline of the log and wake (*y*/*B* = 0). The lateral profiles also confirmed the results shown in Fig. 5 that *k*_*t*_ increased with increasing log length *L*, and that *k*_*t*_ was ten times higher for emergent logs (grey and black symbols in Fig. 6c), compared to submerged logs (white symbols in Fig. 6c). For logs positioned at the sidewall (Fig. 6f), the turbulence within the wake was significantly diminished and more uniform compared to the logs positioned at the channel center (Fig. 6c). In addition, at the edge of the sidewall wake (*y*’/*B* = 0.2, Fig. 6c), the turbulence dropped off more rapidly to approach flow (AF) conditions (crosses in Fig. 6f), for the submerged sidewall log (S5s) than for the emergent sidewall log (S2e), which mirrored the sharper spatial transition in time-mean velocity (Fig. 6d). Both the time-mean and turbulent profiles with sidewall logs (Fig. 6d,f) showed that the submerged log produced a narrower wake than the emergent log.

### Implications for river restoration using wood

**Figure 7:**
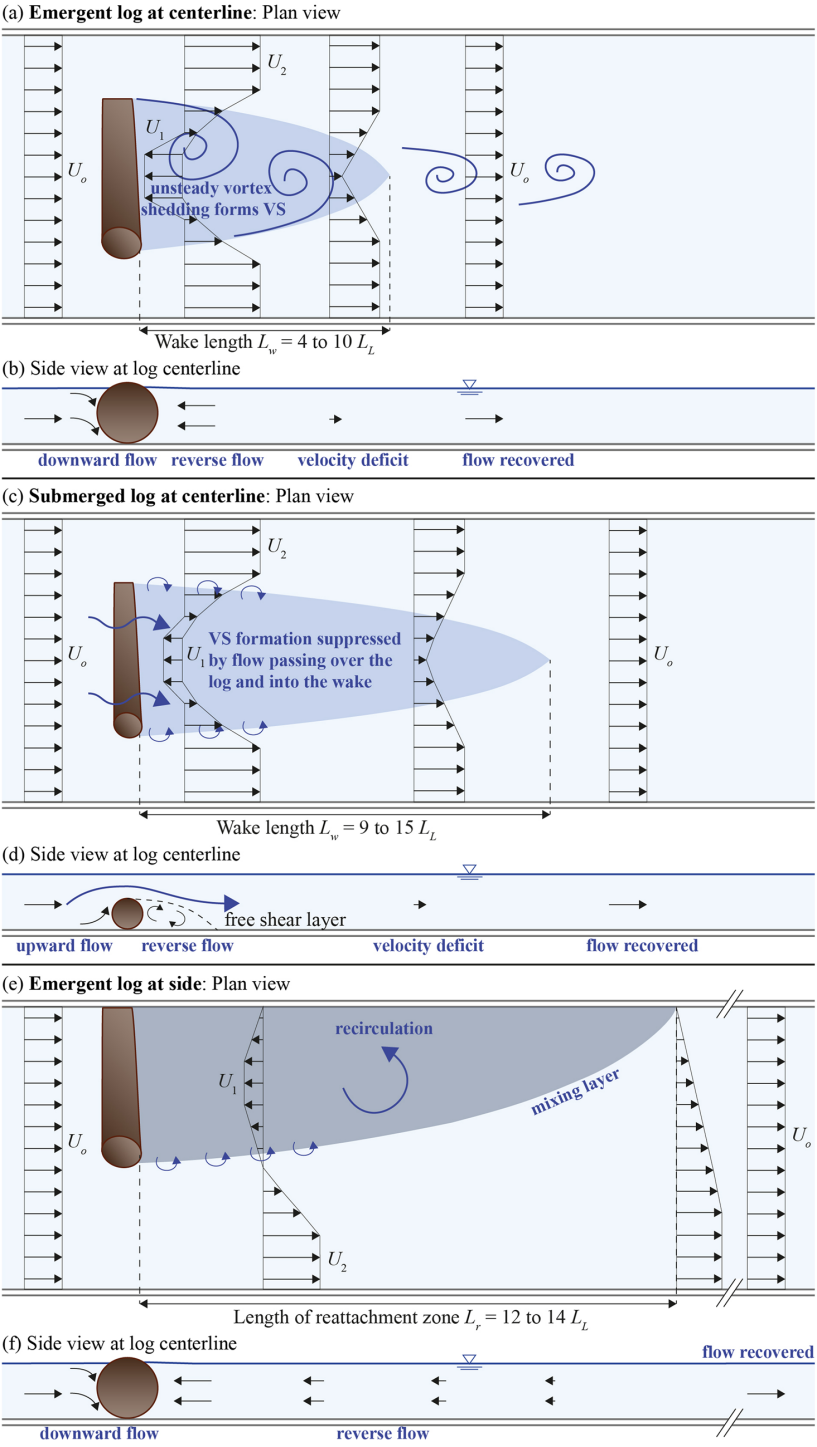
Illustration of resulting flow structures for different log placements; (**a**) emergent log at centerline, (**b**) submerged log at centerline, and (**c**) emergent log at side. VS = von-Kármán vortex street.

The three log placements differently impacted the velocity field (Fig. 7). Emergent logs placed at the channel center produced the highest turbulent kinetic energy, *k*_*t*_, and the strongest recirculation (negative velocity) downstream of the log (Fig. 7a). In contrast, submerged center logs and sidewall logs produced wakes with ten times smaller *k*_*t*_ (Fig. 7b,c), but these wakes were significantly longer in the streamwise extent (Fig. 7b,c). First, consider the impact of the wake on deposition. Similar to the wake behind other vegetation patches, a log wake may act as a region of enhanced deposition of fine particles, such as organic matter and nutrients, that in turn could promote vegetation growth^18,62^. Considering the wakes behind patches of vegetation, Shi et al.^62^ described the preferential accumulation of fine particles in the wake as a function of the channel shear velocity u_*_ and the critical shear velocity u_*c_, which is a function of sediment size. Specifically, deposition was enhanced in the wake, relative to the adjacent channel, only for u_*_/u_*c_ = 0.7 to 3. In this range, resuspension in the main channel provided material to the wake, and the velocity reduction in the wake was sufficient to promote the retention of this material. We anticipate that a similar range of flow conditions would also promote deposition in the wake of a log. Given three different particles with diameters *d*_*m*1_ = 24 μm, *d*_*m*2_ = 0.63 mm (sand), and *d*_*m*3_ = 6.3 mm (gravel), we can estimate the critical shear velocity u_*c_ from the critical Shields parameter *θ*_*c*_ = 0.047 to (e.g., Julien^52^)

5 $$u_{{*c}} = \sqrt {\frac{{\theta _{c} g\left( {\rho _{s} - \rho } \right)d_{m} }}{\rho }} ,$$

with *ρ*_*s*_ = 2650 kg/m^3^, resulting in u_*c1_ = 4.3 mm/s, u_*c2_ = 21.9 mm/s, and u_*c3_ = 69.2 mm/s. The shear velocity of the present experiments can be upscaled to field conditions with λ = 5 to u_*f_ = 8.8 mm/s. We then obtained u_*_/u_*c_ = 2.1 for *d*_*m*1_, u_*_/u_*c_ = 0.4 for *d*_*m*2_, and u_*_/u_*c_ = 0.1 for *d*_*m*3_. Preferential accumulation of fine particles in the log wake would, therefore, occur for *d*_*m*1_. For *d*_*m*2_ and *d*_*m*3_, the channel shear velocity is too low compared to the critical velocity, corresponding to uniform high net deposition in the entire channel.

Second, consider the potential for the log to create fish habitat. This depends on both the size of the eddies formed in the wake and the associated turbulence magnitude. Previous studies suggest that the swimming capabilities of fish are reduced if the eddy size is larger than 75% of the fish length^40,41,53^. As an illustration, let’s compare eddy size to a fish length of 0.2 m^63^ for juvenile (salmonids or cyprinids) and 0.7 m^63^ for adult fish. Focusing on the 0.5 m experimental log, the measured eddy scales (Table 1) were upscaled to the field with a scale factor of λ = 5, i.e., field log length *L*_*f*_  = 2.5 m. For an emergent center log, the VS eddies scale with the log length, 2.5 m, which would exceed 75% of both the juvenile and adult fish lengths, suggesting poor habitat. However, if the center log was submerged, $\overline{{\Lambda_x}}$ = 0.3 ± 0.2 m, which would be smaller than 75% of the adult fish length, but larger than 75% of the juvenile fish length, i.e., providing good habitat for adult fish only. For a log placed at the side, $\overline{{\Lambda_x}}$ = 1.1 m for an emergent log and $\overline{{\Lambda_x}}$ = 0.2 ± 0.2 m for a submerged log. The emergent log would be unsuitable for both adult and juvenile, and the submerged log would be suitable only for adult fish. However, previous studies^41,53,63^ have noted that, in addition to eddy size, turbulence magnitude is also important for fish habitat, and this might expand the suitability of the side-log and submerged-log conditions. For example, emergent center logs, which produce a VS, increased the turbulence within the wake by a factor of 167 above the background turbulence in the open channel (Fig. 5). In contrast, submerged-center logs and both submerged and emergent side logs, none of which produce a VS, only elevated turbulence by a factor of 15 above the background. This difference in turbulence level will affect the fish habitat. Emergent logs may establish a downstream flow region that is preferred by larger or adult fish due to the larger eddies, stronger turbulence, and higher drift densities due to increased velocity next to the log, while submerged or side logs may create flow regions preferred by smaller or juvenile fish with smaller eddies and weaker turbulence^38–40,63^. This illustrates how by varying log sizes and degree of submergence, habitat suitability for different fish preferences can be created.

## Summary

The wake and flow structures associated with different log placement were experimentally investigated. For emergent logs placed at the channel center, a von-Kármán vortex street (VS) was formed, characterized by the unsteady detachment of vortices from alternating ends of the log, which subsequently migrated downstream. In contrast, no VS was formed for submerged logs, due to the suppression of vortex formation by the flow passing over the top of the log and into the wake. The formation of a VS elevated the turbulence within the wake by a factor of 10, compared to the turbulence observed without VS formation. Due to their significantly lower turbulent mixing, the wakes behind submerged center logs required a much greater distance to exchange momentum with the outer flow and recover velocity within the wake. As a result, the wakes generated by submerged center logs were two to three times longer than the wake generated by emergent center logs. We further observed a hybrid case for *h*/*d* = 1.17 with two peaks in *k*_*t*_, with the first peak associated with the increased flow above the log (sweeping flow), and the second, smaller peak present due to the lateral shear. Logs positioned at the channel sidewall had the lowest turbulence levels and greatest streamwise extent, which can increase the residence time and deposition of fine particles such as organic matter and nutrients. Finally, the eddy scale and turbulence magnitude in the wakes depend on the log size, placement and submergence, which can be selected to target specific species and fish ages. Given the heterogeneity of piece size, location, orientation, and submergence of naturally occurring large wood in rivers^63^, we suggest that river restoration would benefit from equally diverse characteristics in deliberately placed large wood in order to maximize habitat diversity in rivers.

## Supplementary Information

Supplementary Information

## Data Availability

Data sets for this research are available at 10.5281/zenodo.4665770.
