# Supplementary material for: Flow and wake characteristics associated with large wood to inform river restoration
Source: Sci Rep. 2021 Apr 21;11:8644. doi: 10.1038/s41598-021-87892-7 (PMC8060320; doi:10.1038/s41598-021-87892-7)
Supplement: Supplementary file 1 — Supplementary Information [file 41598_2021_87892_MOESM1_ESM.pdf]

Flow and wake characteristics associated with large wood to inform river restoration

Isabella Schalko[1,2*], Ellen Wohl[3] & Heidi M. Nepf[1]

[1]Department of Civil and Environmental Engineering, Massachusetts Institute of Technology, Cambridge, MA, USA. [2]Laboratory of Hydraulics, Hydrology and Glaciology, ETH Zurich, Zurich, Switzerland. [3]Department of Geosciences, Colorado State University, Fort Collins, CO, USA. *email: ischalko@mit.edu

Supplementary information

Integral length scale

The integral length scale Λ_x, a measure of the largest eddy size in a turbulent flow, was determined at multiple points in the wake using the autocorrelation function of the streamwise velocity measured at the log centerline at $z = d/2$:

$$\Lambda_x = \bar{u} \int_0^{\tau_0} \frac{\overline{u'(t)u'(t+\tau)}}{\overline{u'^2}} \mathrm{d}\tau. \tag{S1}$$

Here, τ is the time lag with respect to t and τ_0 is τ at $\Lambda_x = 1/\mathrm{e}$. The variance-normalized autocorrelation function of each $u'(t)$ record was calculated using the xcov.m function in Matlab. This procedure was applied only for data points that fulfilled the following two conditions: (1) the time-averaged velocity $\bar{u} > 0$ (i.e., not in regions with flow recirculation) and (2) the turbulence strength, u_{rms}, was small compared to the time-averaged flow velocity, $\bar{u}$, i.e., $\frac{u_{rms}}{\bar{u}} < 0.10$, which supports the frozen turbulence hypothesis used in (S1). For tests without VS (C4s-C7s; S1e-S5s), $\bar{u}$ in (S1) was the time-averaged velocity at the point at which the autocorrelation was evaluated. Vortices in a von-Kármán vortex street convect at the speed of the open channel adjacent to the wake. When a vortex street (VS) was present and unconfined (C3e), $\bar{u}$ in (S1) was defined as the channel-average upstream velocity. When VS was present but influenced by the confinement of the walls (C1e-C2s), $\bar{u}$ in (S1) was assumed to be the velocity adjacent to the wake.

In addition, for tests with an observed VS (C1e-C3e), a peak integral length scale Λ_{peak} was also calculated from the observed peak frequency $f_{peak,\,v}$ in the power spectra of the lateral velocity component S_{vv} (Figure 4; main text).

$$\Lambda_{peak} = \frac{\bar{u}}{(2\pi f_{peak,\,v})}, \tag{S2}$$

with $\bar{u}$ defined as the channel-average upstream velocity for unconfined wakes (C3e) and the velocity adjacent to the log for confined wakes (C1e-C2e). This length-scale is the most direct measure of the von-Kármán vortices, and it scales with the log length L. The values are listed

in Table 1 of the main text. In Figure S1, Λ_x are plotted for all tests satisfying the constraints to apply (S1) along the log centerline.

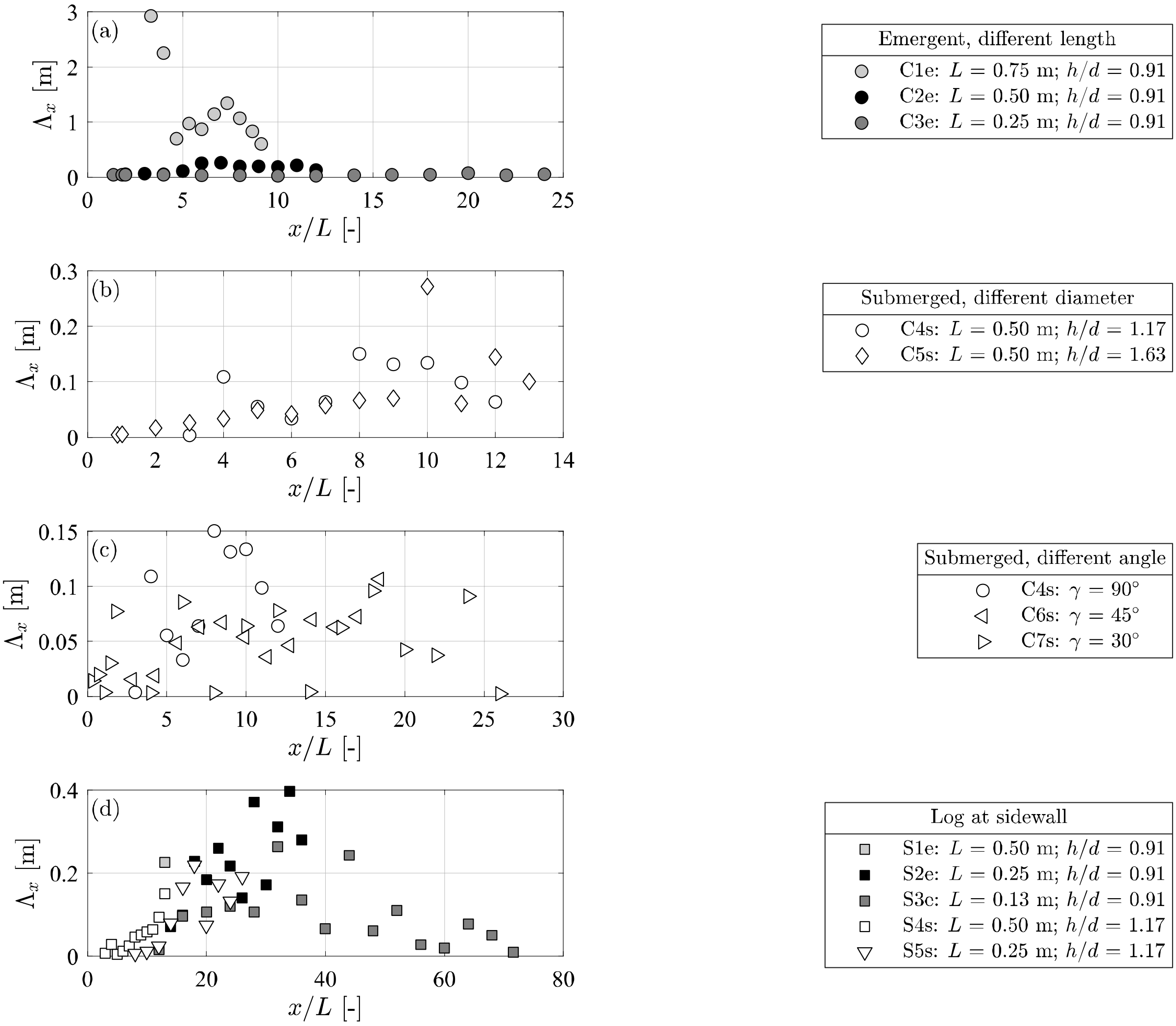

Figure S1. Longitudinal profile of integral length scale Λ_x in x-direction for all tests.
